# The Intestinal Bacterial Community and Functional Potential of *Litopenaeus vannamei* in the Coastal Areas of China

**DOI:** 10.3390/microorganisms9091793

**Published:** 2021-08-24

**Authors:** Yimeng Cheng, Chaorong Ge, Wei Li, Huaiying Yao

**Affiliations:** 1 Research Center for Environmental Ecology and Engineering, School of Environmental Ecology and Biological Engineering, Wuhan Institute of Technology, Wuhan 430073, China; ymcheng@126.com (Y.C.); mmli234@163.com (W.L.); hyyao@iue.ac.cn (H.Y.); 2 Zhejiang Key Laboratory of Urban Environmental Processes and Pollution Control, Ningbo Urban Environment Observation and Research Station, Chinese Academy of Sciences, Ningbo 315800, China; 3 Key Laboratory of Urban Environment and Health, Institute of Urban Environment, Chinese Academy of Sciences, Xiamen 361021, China

**Keywords:** *Litopenaeus vannamei*, intestinal bacterium, functional potential, potential pathogenic bacteria and spoilage organisms, Chinese coastal areas

## Abstract

Intestinal bacteria are crucial for the healthy aquaculture of *Litopenaeus vannamei*, and the coastal areas of China are important areas for concentrated *L. vannamei* cultivation. In this study, we evaluated different compositions and structures, key roles, and functional potentials of the intestinal bacterial community of *L. vannamei* shrimp collected in 12 Chinese coastal cities and investigated the correlation between the intestinal bacteria and functional potentials. The dominant bacteria in the shrimp intestines included *Proteobacteria*, *Bacteroidetes*, *Tenericutes*, *Firmicutes,* and *Actinobacteria*, and the main potential functions were metabolism, genetic information processing, and environmental information processing. Although the composition and structure of the intestinal bacterial community, potential pathogenic bacteria, and spoilage organisms varied from region to region, the functional potentials were homeostatic and significantly (*p* < 0.05) correlated with intestinal bacteria (at the family level) to different degrees. The correlation between intestinal bacteria and functional potentials further suggested that *L. vannamei* had sufficient functional redundancy to maintain its own health. These findings help us understand differences among the intestinal bacterial communities of *L. vannamei* cultivated in different regions and provide a basis for the disease management and healthy aquaculture of *L. vannamei*.

## 1. Introduction

*Litopenaeus vannamei* has a thin shell, substantial meat and provides an excellent source of nutrients such as protein, nonprotein nitrogen compounds (amino acids), and unsaturated fatty acids. Therefore, customers widely favor *L. vannamei* is widely [1,2,3]. *L. vannamei* is one of the most cultivated species worldwide and the most commonly cultivated marine organism in China [4,5] with numerous advantages such as rapid growth, short maturation period, low nutritional requirements and strong adaptability to the environment. However, for the continued expansion of the aquaculture scale [6], improvements in intensive aquaculture are needed [7]. However, the rapid deterioration of aquaculture environments [8,9,10] and frequently occurring aquaculture diseases [11,12,13] are important factors in restricting and hindering the development of the aquaculture industry [14]. The safety of *L. vannamei* products is closely related to human health [12,13,15,16]. For example, aquatic catches, such as *L. vannamei*, may deteriorate during fishing and sales, affecting the quality of the associated products and causing potential food health risks [1,17]. Therefore, frequent disease occurrence and food safety in *L. vannamei* aquaculture have received increased attention [1].

Understanding the mechanisms and causes of aquaculture diseases is crucial to ensure food health and avoid food safety risks during *L. vannamei* preservation. *L. vannamei* diseases are primarily caused by bacteria and viruses [18], but fungi, parasites, *Rickettsiella,* and other pathogens also cause disease in *L. vannamei* [14,19]. For example, *white spot syndrome virus* (WSSV) [11], *yellow head virus* (YHV) [20], *monodon baculovirus* (MBV) [21,22], *Taura syndrome virus* (TSV) [23], *hepatopancreatic parvovirus* (HPV) [24], *infectious hypodermal, and hematopoietic necrosis virus* (IHHNV) [24] are viruses of *Penaeus.* Notably, some clinical pathogenic *Vibrio* species, such as *Vibrio cholerae* [25], *Vibrio parahaemolyticus* [12,13] and *Vibrio vulnificus* [26,27], which exist in or parasitize the tissues of *L. vannamei*, are also important human pathogenic bacteria [28,29,30,31,32]. Many studies have shown that intestinal bacteria influence several aspects of animal health [33], including feeding, growth, pathogen defense, energy metabolism, reproduction, immunity, and aging [34], in crucial ways. Specifically, the intestinal microbiota plays an important role in attaining essential nutrient resources for arthropods [35,36]. Functional and biological characteristics also vary with changes in intestinal microbial community composition [37,38,39]. Therefore, most of the conditional pathogens that parasitize the intestine [31] are symbiotic with other beneficial bacteria of *L. vannamei,* including normal microflora [40], which may play a dominant role in ensuring that the intestinal ecosystem possesses sufficient functional redundancy to maintain gastrointestinal health [36,41,42]. However, the biological environment of *L. vannamei* is affected when the environment and culture mode is changed or destroyed [43]; the balance of the symbiosis between opportunistic pathogenic bacteria and other beneficial bacteria is disrupted, leading to various diseases of *L. vannamei* [44]. The microbial system, especially the intestinal microbial system of *L. vannamei*, varies with the living environment and conditions, including changes in the region, feed, and species, leading to different degrees of spoilage and food quality degradation even under the same storage mode [45,46,47]. As a result, the safety and storage methods for *L. vannamei* deriving from different regions and cultured under different environmental conditions are also very different [48]. Therefore, it is necessary to understand the differences between and composition of *L. vannamei* intestinal microorganisms deriving from different regions and cultured under different environmental conditions to provide new strategies for safe and healthy aquaculture and the preservation and consumption of *L. vannamei.* The output of *L. vannamei* cultured in seawater from the coastal areas of China, an important area of concentrated *L. vannamei* aquaculture, reached 1.14 million tons in 2019 [49]. Some of the bacteria associated with shrimp diseases are of concern [12,50,51]. Conversely, studies on the intestinal microflora of healthy *L. vannamei* under different culture conditions and growth stages have also been performed [52,53]. However, the differences in and comparisons between the intestinal microflora of healthy *L. vannamei* in coastal areas of China are not known.

The intestinal bacterial community structure, composition, and differences in the commercial and healthy *L. vannamei* cultivated in 12 Chinese coastal cities were investigated using bacterial 16S rRNA gene high-throughput sequencing. Furthermore, the distribution and existence of pathogenic bacteria and spoilage organisms were examined in the intestine of *L. vannamei* shrimp from 12 different regions. The findings help elucidate the distribution of intestinal bacterial communities of *L. vannamei* in coastal areas of China and provide a theoretical basis for microecologically healthy aquaculture and consumption of *L. vannamei*.

## 2. Materials and Methods

### 2.1. L. vannamei Procurement and Intestinal Sample Collection

*L. vannamei* was obtained from local aquaculture farms from 12 cities along the coast of China across nine Chinese provinces during September 2020 (accessions provided in Table S1). The seawater temperature was relatively stable at 22.6–30.4 °C, and the pH value ranged from 7.7–8.3 (Table S2). Several evenly sized (11.0 ± 0.2 cm) and healthy *L. vannamei* shrimp were collected from each city and quickly frozen at −40 °C after being caught. They were frozen overnight, then shipped individually with dry ice and ice bags to the laboratory. The frozen *L. vannamei* shrimp naturally thawed at 0 °C, and each thawed shrimp was dissected using sterile forceps and tweezers to obtain intact intestines under sterile conditions. The intestinal sample was used for intestinal microbial analysis to determine the interindividual variation in intestinal microbiota. The intestines of three shrimp were pooled as one sample, with three samples in total per group. For each city, three dissected intestines were placed into a 2 mL Eppendorf tube and set as one repeat intestinal sample. Each intestinal samples used three replicates, resulting in a total of 36 samples for the 12 cities. The resulting intestinal samples were preserved at −80 °C until DNA extraction.

### 2.2. L. vannamei Intestinal DNA Extraction

Total DNA was extracted from intestinal samples using a FastDNA Spin Kit for Feces (MP Biomedicals, Illkirch, France), and the DNA isolation procedure was generally performed according to the manufacturer’s instructions. Briefly, the intestinal samples were successively homogenized by FastPrep-24™ 5G (MP Biomedicals, Illkirch, France), and proteins were precipitated using a PPS solution in a refrigerated centrifuge. The DNA was attached to a SPIN™ filter tube (MP Biomedicals, Illkirch, France), and the SPIN™ filter column was washed and dried. Immediately after this procedure, the extracted intestinal DNA was eluted using 100 μL of DES solution, and the concentration and quality of extracted DNA were checked using 1.0% agarose gel electrophoresis and spectrophotometric analysis (Nanodrop ND-1000, Thermo Fisher Scientific, Waltham, MA, USA). The extracted DNA was stored at −20 °C for further analysis.

### 2.3. 16S rRNA Gene Amplification, Illumina Sequencing and Bioinformatics Analysis

To target the hypervariable V3−V4 region of the bacterial 16S rRNA gene, the universal primers 341F (5′-CCT AYG GGR BGC ASC AG-3′) and 806R (5′-GGA CTA CNN GGG TAT CTA AT-3′) were used to amplify the intestinal DNA samples. A 20 μL reaction, containing 4 μL of 5× Fast Pfu Buffer, 2 μL of 2.5 mM dNTPs, 0.8 μL of forward primer (5 μM), 0.8 μL of reverse primer (5 μM), 0.4 μL of FastPfu Polymerase, 0.2 μL of BSA, and 10 ng of template DNA and ultraclean water, was performed in a PCR System (GeneAmp^®^9700, ABI) under the following conditions: initial enzyme activation for 3 min at 95 °C; 29 amplification cycles of denaturation for 30 s at 95 °C, annealing for 30 s at 53 °C and an extension for 45 s at 72 °C; a final extension for 10 min at 72 °C, and a 10 °C step maintained until halted by the user. The PCR products were checked and purified using 2.0% agarose gel electrophoresis and a Universal DNA Purification Kit (Tiangen, Beijing, China). The purified PCR products were submitted for sequencing on an Illumina MiSeq platform (Shanghai Majorbio Bio-pharm Technology Co., Ltd., Shanghai, China). The Quantitative Insights into Microbial Ecology (QIIME) process was used to analyze the high-throughput sequencing data and select the operational taxonomic units (OTUs) at the 97% similarity level (Usearch (v.7.1 http://drive5.com/uparse/, accessed on 8 April 2021)). The taxonomy of each representative sequence was assigned via the RDP Classifier based on the Silva v.132 16S rRNA gene database (http://www.arb-silva.de, accessed on 14 April 2021).

### 2.4. Statistical Analysis

A total of nine *L. vannamei* specimens were selected from each region, and one sample used three *L. vannamei* intestines with three replicates, resulting in a total of 36 intestinal DNA samples from 12 cities along the coast of China. All the data were calculated using Microsoft Excel and represented the mean value ± standard error (SE), as shown in the Figures. R v.4.0.5 with vegan 2.5–7 calculated and described the alpha diversity (Chao1 richness estimator and Shannon index) of the intestinal bacteria of *L. vannamei*. The Adonis test and principal coordinates analysis (PCoA) were used to compare the beta diversity of different samples. IBM SPSS v.25 analyzed differences among the samples and associated OTUs using single-factor analysis of variance (ANOVA), the least significant difference (LSD) test, Bayesian model-based moderated tests, and *t*-tests. Significant differences were detected at the 0.05 level. R v.4.0.5 with Tax4Fun2 1.1.3 was used to predict functional profiles and identify the functions of intestinal bacteria based directly on 16S rRNA gene information. OriginPro v.9.8.0.200 (OriginLab Corporation, Northampton, MA, USA) generated the bar charts, presented the correlations of intestinal bacteria (at the family level), and predicted gut microbial functions (relative abundance) based on Pearson’s correlation. This analysis should further expand the understanding of intestinal microbial ecology [54]. R v.4.0.5 with pheatmap 1.0.12 was used to draw heatmaps of intestinal bacterial composition at the family level.

## 3. Results

### 3.1. Diversity of the Intestinal Bacterial Community of L. vannamei from Different Regions

After 16S rRNA high-throughput sequencing, a total of 1,370,304 high-quality sequences were obtained from 36 samples, and 38,064 OTUs were identified based on the QIIME process at the 97% similarity level. Rarefaction curves (Figure S1a) presented the richness of all the samples (label: 0.03) and the Shannon-Wiener index (Figure S1b) assessed diversity.

**Figure 1:**
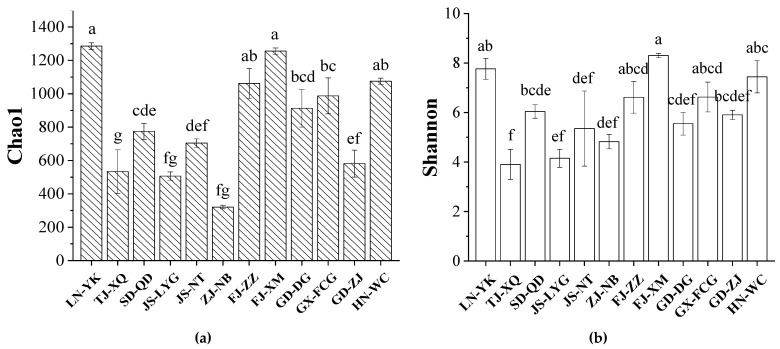
Alpha diversity ((**a**) Chao1 richness estimator, (**b**) Shannon index) of intestinal bacteria among different samples (mean ± SE). Different letters indicate significant differences.

**Figure 2:**
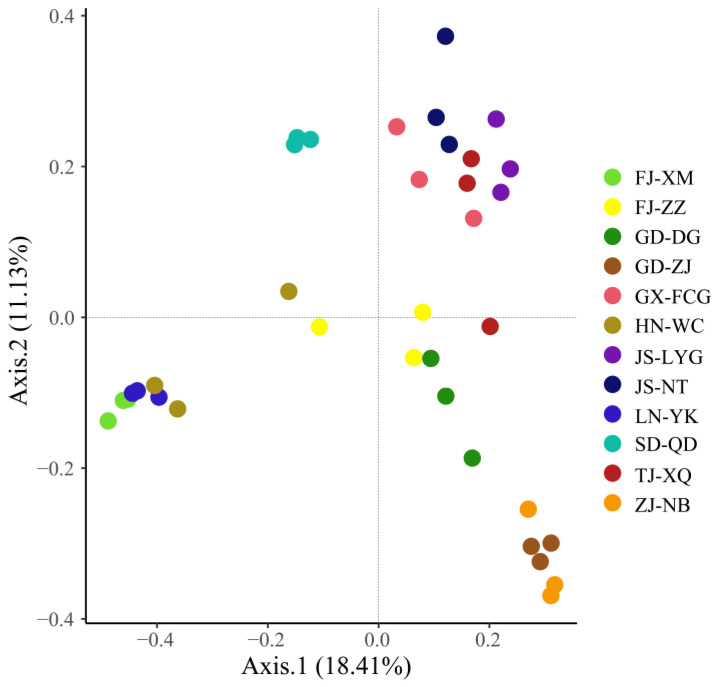
Beta diversity (PCoA of the intestinal bacterial community at the OTU level) of intestinal bacteria among different samples.

The intestinal bacterial community diversity of *L. vannamei* was analyzed by the Chao 1 estimator (Figure 1a) and the Shannon index (Figure 1b). The results showed that the intestinal bacterial community richness and diversity varied in different areas on the coast of China. For example, there were significant differences (*p* < 0.001) in intestinal bacterial diversity among different samples (LN-YK vs. TJ-XQ, LN-YK vs. JS-LYG, TJ-XQ vs. FJ-XM, TJ-XQ vs. HN-WC). PCoA of the intestinal bacterial community at the OTU level was used to visualize differences in the intestinal bacterial community of *L. vannamei* from 12 different regions of China’s coastal area (Figure 2). Samples of LN-YK, FJ-XM, and HN-WC, samples of GX-FCG, JS-LYG, TJ-XQ, and JS-NT, and samples of ZJ-NB and GD-ZJ were separated into clusters. The SD-QD samples were significantly clustered together and differed from the other regional samples (Adonis test, *p* < 0.001). The diversity and similarity distribution of intestinal bacterial communities from different coastal areas were not significantly related to the geographical location. In addition, although the pH of seawater (Table S2) in was different between the regions, there was no significant correlation between *L. vannamei* intestinal microflora and pH value (as determined by ANOVA, *p* = 0.30, R = 0.18).

### 3.2. Composition of the Intestinal Bacterial Community of L. vannamei from Different Regions

#### 3.2.1. Composition of the Gut Microbiota

High-throughput sequencing showed that the intestinal bacterial community composition and structure at the phylum level varied from region to region (Figure S2). The changes and differences in the relative abundances of *Proteobacteria*, *Bacteroidetes*, *Tenericutes*, *Firmicutes,* and *Actinobacteria* were the most significant among different areas. The dominant phyla in the intestine of *L. vannamei* shrimp from different regions also showed differences. For example, *Tenericutes* was the dominant phylum in JS-NT and JS-LYG (35.4–38.1%); *Firmicutes* was the dominant phylum in TJ-XQ and JS-LYG (38.0–53.4%), and *Actinobacteria* was the dominant phylum in SD-QD (31.9%).

**Figure 3:**
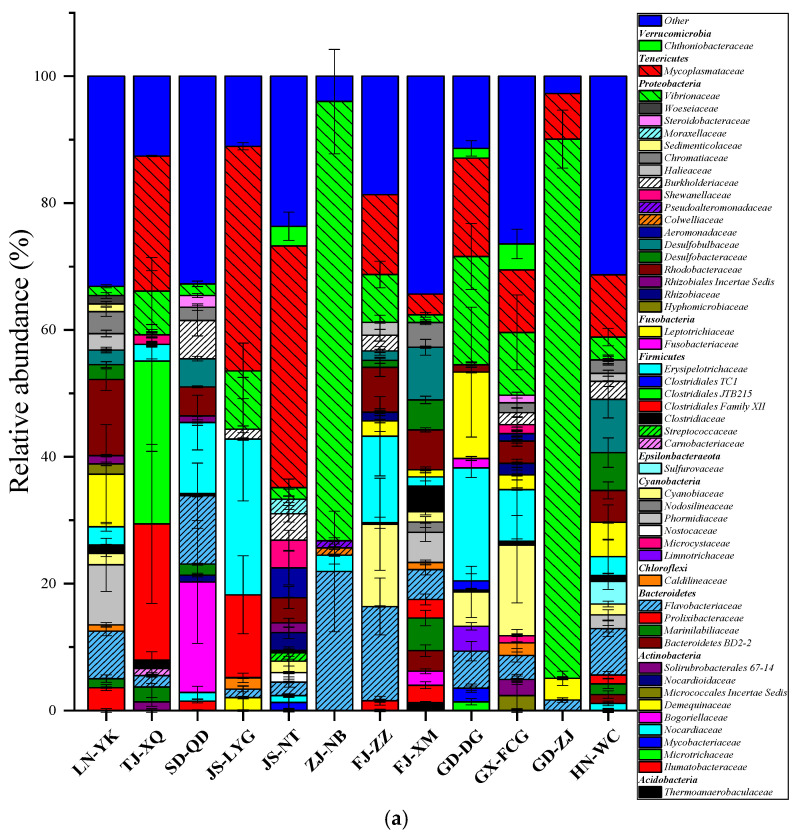

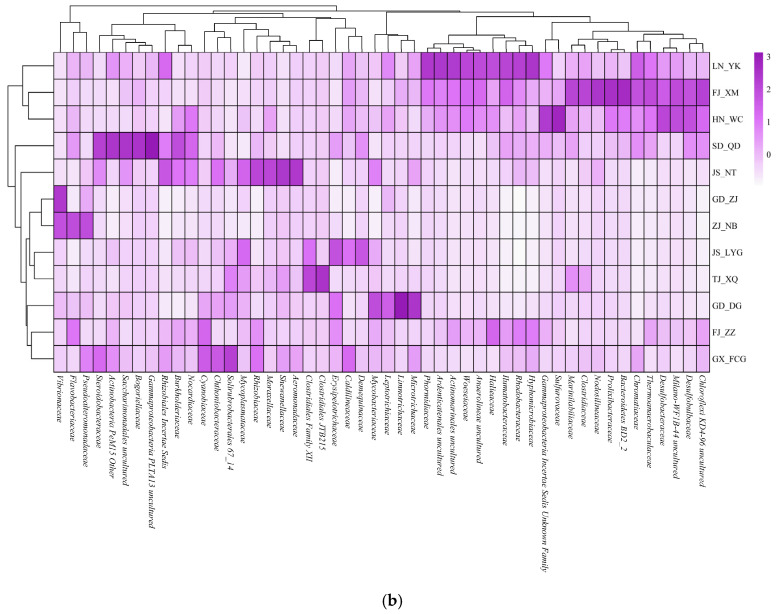
Composition of the intestinal bacterial community of *L. vannamei*. (**a**) Only families with an average relative abundance >1% are shown here (see Table S1 for abbreviations). The relative abundance of each family in the same group is indicated by color intensity. (**b**) Heat map showing the distribution of the top 50 abundant families in the intestinal bacterial communities. Relative abundance percentage values for the bacterial families are indicated by color intensity.

At the family level, the composition of the intestinal bacteria varied more apparently among regions (Figure 3a). As shown by the heat map of the top 50 abundant families in the intestinal bacterial communities (Figure 3b), the composition and relative abundances of dominant taxa in the different samples were also different. Although *Vibrionaceae*, *Mycoplasmataceae*, *Erysipelotrichaceae,* and *Flavobacteriaceae* were the most abundant taxa and played a certain dominant role in the intestinal bacterial community of all the samples, *Rhodobacteraceae* (12.0%), *Clostridiales JTB215* (25.7%), *Bogoriellaceae* (17.4%), *Flavobacteriaceae* (14.9%), *Erysipelotrichaceae* (17.8%), and *Cyanobiaceae* (14.3%) were the dominant taxa in the LN-YK, TJ-XQ, SD-QD, FJ-ZZ, GD-DG, and GX-FCG samples, respectively. Notably, the relative abundance of *Mycoplasmataceae* was the highest in JS-LYG, JS-NT, and HN-WC, reaching 35.4–38.1%. The relative abundance of *Vibrionaceae* in ZJ-NB and GD-ZJ reached 69.2–85.0% (Table S3).

Among all the intestinal samples of *L. vannamei* from 12 coastal cities of China, 20 common taxa were found (Table S4). When the bacterial composition at the family level of all samples was compared, only *Gammaproteobacteria EV818SWSAP88 uncultured bacterium*, *RsaHF231 uncultured bacterium,* and *Gammaproteobacteria MBMPE27 uncultured bacterium*, with low relative abundances, were found in TJ-XQ, GD-DG, GX-FCG, and HN-WC, respectively. *Schekmanbacteria Other* was uniquely present in the FJ-ZZ sample, and seven unique taxa, namely, *Acidimicrobiia IMCC26256 uncultured bacterium*, *Chitinophagaceae*, *Sphingobacteriaceae*, *Simkaniaceae*, *Roseiflexaceae*, *Wohlfahrtiimonadaceae,* and *Cellvibrionaceae*, were found only in the JS-NT samples (Figure S3).

#### 3.2.2. Potential Pathogen and Spoilage Organisms in the Intestine of *L. vannamei*

The microbial system, especially the intestinal microbial system of *L. vannamei*, varies based on the living environment and conditions, including region, feed, and species [45,46,47]. The potential pathogenic bacteria and spoilage organisms parasitizing the intestine reflect the microecological health of the culture and may become standards for food health sub-risk assessment of *L. vannamei* [44].

**Figure 4:**
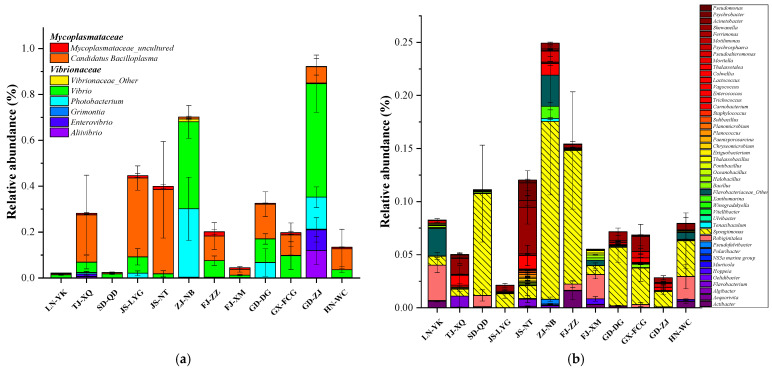
Relative abundances (at the genus level) of potential pathogenic bacteria (**a**) and spoilage organisms (**b**) in the intestine of *L. vannamei* (mean, *n* = 3).

Potential pathogenic bacteria and spoilage organisms, which are often considered and investigated in the tissues of *L. vannamei* [31,51], were also present at relatively high abundances in all the samples and were screened and analyzed statistically (Figure 4a for potential pathogenic bacteria and Figure 4b spoilage organisms). The samples from each area contained a certain amount of potential pathogenic bacteria and spoilage organisms, and the composition and relative abundances of these organisms in the intestine of *L. vannamei* varied among different regions. The relative abundance of potential pathogenic bacteria in most samples was above 10%. *Vibrionaceae* was dominant in the ZJ-NB and GD-ZJ samples, and the relative abundance of this taxon reached above 60%. The proportion of *Mycoplasmataceae* in the TJ-XQ, JS-LYG, and JS-NT samples was greater than that of *Vibrionaceae*. The composition of dominant spoilage organisms also differed among regions, and the relative abundance of potential spoilage organisms (at the family level) was above 10% in the SD-QD, JS-NT, ZJ-NB, and FJ-ZZ samples. However, *Spongiimonas* was dominant (2.3–3.3%) in LN-YK, and FJ-XM, and *Shewanella* was dominant (1.5–4.4%) in TJ-XQ and JS-NT.

### 3.3. Prediction of Gut Microbial Functions

**Figure 5:**
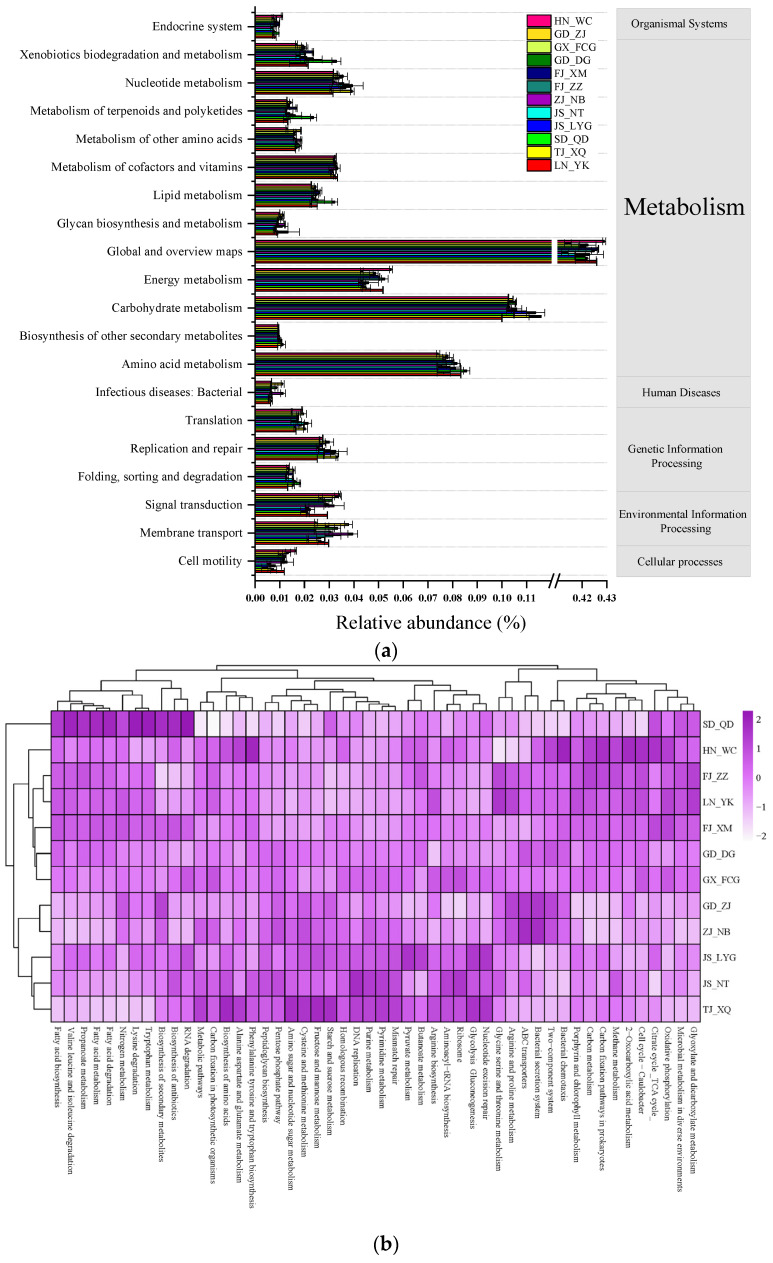
Predictive functional profiling of the bacterial communities in the gut of *L. vannamei* analyzed by Tax4Fun based on the KEGG database (mean, *n* = 3). (**a**) Only genes with an average relative abundance (at level 2) >1% are shown here. (**b**) Heat map showing the predictive functional profiling of the top 50 KEGG pathways (at level 3). Relative percentage values for the KEGG pathways are indicated by color intensity.

The potential functions of the microbial community in the intestine of *L. vannamei* were predicted using Tax4Fun2 based on the KEGG database. The major functions predicted were involved in organismal systems (1.47–1.98%), metabolism (79.28–84.89%), human diseases (1.99–3.45%), genetic information processing (5.40–8.45%), environmental information processing (3.85–7.97%) and cellular processes (0.96–2.73%) (Figure 5a and Figure S4). There were eight pathways for organismal systems, twelve pathways for metabolism, eleven pathways for human diseases, four pathways for genetic information processing, three pathways for environmental information processing, and four pathways for cellular processes. Generally, the abundances of these individual functional categories and groups were close to each other among the twelve coastline cities in China, which suggests a relatively stable survival and adaptation strategy for microbial community function during the growth and transport of *L. vannamei*.

For the metabolism category, global and overview maps (41.3–42.9%), carbohydrate metabolism (10.0–11.6%), and amino acid metabolism (7.4–8.6%) were the abundant pathways. Frequently occurring gene types in environmental information processing were involved in membrane transport and signal transduction, and the gene types in genetic information processing were involved in replication, repair and translation.

For the SD-QD samples, the relative abundances of genes related to RNA degradation, biosynthesis of antibiotics, biosynthesis of secondary metabolites, tryptophan metabolism, lysine degradation, nitrogen metabolism, fatty acid degradation, fatty acid metabolism, propanoate metabolism, valine, leucine, and isoleucine degradation and fatty acid biosynthesis, were the most abundant (Figure 5b). The potential functional profiles also showed clustering phenomena in Zhanjiang, Guangdong, and Ningbo, Zhejiang. The relative abundances of genes associated with two-component systems, ABC transporters, bacterial secretion systems, and arginine and proline metabolism were relatively high.

### 3.4. Correlation of Intestinal Bacteria and the Predicted Functional Profile

**Figure 6:**
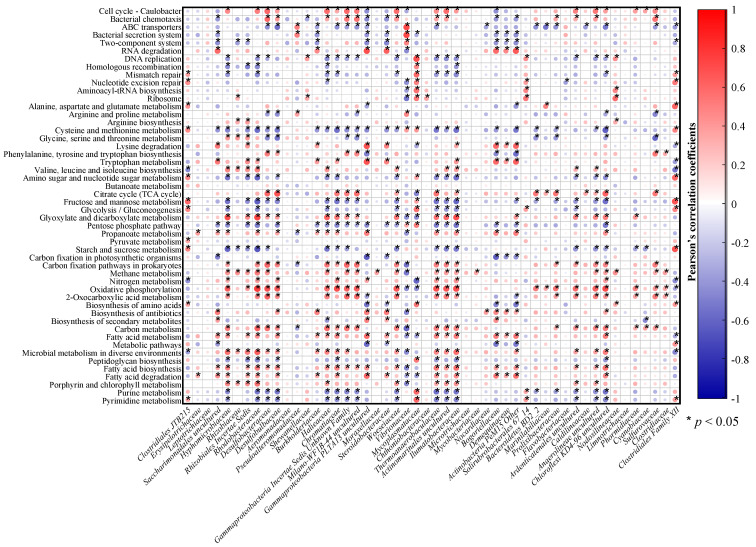
Correlations of intestinal bacteria (at the family level) and predicted gut microbial functions (relative abundances) based on Pearson’s correlation coefficients (*p* < 0.05). The red circles represent a positive correlation, the blue circles represent a negative correlation, and the circle sizes reflect the absolute value of the correlation coefficient.

The relationship between the major KEGG pathways (at level 3) and the dominant flora (at the family level) was analyzed by a correlation plot (OriginPro 2021) (“*”, *p* < 0.05) (Figure 6). Although the bacterial community had functional redundancy, there was a strong positive correlation between the community function and the bacterial community as a whole (red indicates a large proportion). Some flora components had very similar functionally related structures, such as *Hyphomicrobiaceae*, *Rhizobiales Incertae Sedis*, *Rhodobacteraceae*, *Desulfobulbaceae*, *Desulfobacteraceae*, *Halieaceae*, *Chromatiaceae*, *Gammaproteobacteria Incertae Sedis Unknown Family*, *Milano-WF1B-44 uncultured*, *Chthoniobacteraceae*, *Thermoanaerobaculaceae*, *Actinomarinales uncultured,* and *Chloroflexi KD4-96 uncultured*.

A major finding was that several functions showed significant correlations, such as cell cycle—Caulobacter within cell growth and death, citrate cycle (TCA cycle), and glyoxylate and dicarboxylate metabolism within carbohydrate metabolism, carbon fixation pathways in prokaryotes and oxidative phosphorylation within energy metabolism, and 2-oxocarboxylic acid metabolism, carbon metabolism, and fatty acid metabolism within global and overview maps.

## 4. Discussion

The relationship between intestinal microorganisms and the host has attracted much attention due to its specific products or for disease prevention. The differences in intestinal microbiota structure and composition may affect many important physiological activities of the host, such as nutrient processing, energy balance, immune function, and development [35,36,37,38,39]. Specifically, intestinal microflora is closely related to physiological processes and plays an important role in the growth of *L. vannamei*, an important factor in maintaining the stability of the intestinal environment [55,56]. *L. vannamei* relies mainly on various types of nonspecific immune factors to recognize alien organisms and resist environmental stress and infection by various pathogens due to the lack of an acquired immune system [57]. Therefore, the structure and function of the normal intestinal bacterial community enhances the body’s immune function and provides a powerful barrier for the prevention and control of pathogenic bacteria with the intestinal mucosa, which further hinders invasion by pathogenic bacteria and maintains the stability of the organismal environment. The diversity, structure, composition, and functional potential of the intestinal microbial community may vary based on the environment, such as the organic and inorganic compounds in water, nutrients in feed, parasites, and microorganisms in the culture environment [36,58]. Therefore, high-throughput sequencing and bioinformatics analysis showed that the diversity, structure, and composition of the intestinal microbial community of *L. vannamei* from 12 Chinese coastal cities all varied from region to region and were not significantly related to geographical location. However, the species specificity of microorganisms enables closely related bacteria from the same taxa and different taxa to form communities in a particular environment via quorum sensing due to their similar or complementary functions [59]. Therefore, the functional potentials were relatively similar among all of the samples. This relationship may represent the functional redundancy formed by *L. vannamei* to resist different environmental stresses and ensure their own gastrointestinal health [36].

The intestinal microorganisms of *L. vannamei* species are complex, variable, and crucial to the health and physiological activities of the host. They play a pivotal role in growth, digestion, absorption, immune disease prevention, and other processes in these organisms. The complex and variable intestinal flora is a balanced system formed by interactions among the host, environment, and microorganisms [60]. However, the intestinal microbial dynamic balance of *L. vannamei* is affected by many factors, including host factors (genetics, physiology, growth, development, etc.) and nonhost factors (feed, aquaculture environment conditions, exogenous microorganisms or compounds, etc.) [61,62]. For host factors, there are some genetic differences in *L. vannamei* that may lead to different tissue structures and functional habitats. The structure and function of bacteria adhering to the intestinal mucosa and parasitizing the intestine may differ due to differences in physicochemical properties and nutrients in the intestinal environment [63,64]. Regarding nonhost factors, the water environment, food, and exogenous material also strongly affect the intestinal microbial community of aquatic animals [53,65]. No significant correlation between the intestinal microflora and the pH value of water quality further proved that the intestinal microflora was affected by a variety of factors. For *L. vannamei*, these host and nonhost factors cause differences in the intestinal microorganisms at different developmental stages and lead to differences in the intestinal microorganisms in different culture areas. These differences between *L. vannamei* shrimp from different regions ensure that the intestinal micro-organisms complete signal transduction and physiological metabolic functions. Although there were differences in the relative abundance of *L. vannamei* intestinal microorganisms from 12 Chinese coastal cities, there were also commonalities in the structure and composition of the microbial communities (Figure 3 and Figure S3). *Proteobacteria*, *Bacteroidetes*, *Tenericutes*, *Firmicutes,* and *Actinobacteria* dominated the *L. vannamei* intestinal microbiota, similar to previous reports [66,67]. 

As previously reported, potential pathogenic bacteria primarily cause bacterial diseases in *L. vannamei* [68,69], and disease outbreaks persist despite many preventive efforts [70,71]. Some clinical pathogenic *Vibrio* species, such as *V. cholerae* [25], *V. parahaemolyticus* [12,13], and *V. vulnificus* [26,27], which parasitize or live in the tissues of *L. vannamei*, are also important human pathogenic bacteria [28,29,30,31,32]. The spoilage organisms that parasitize the intestine of *L. vannamei* should also be considered for their potential threat to food health in humans and product quality [32,72,73]. As previously reported, the structure and abundance of intestinal microorganisms remain in dynamic balance under normal physiological conditions. However, imbalance may occur when normal flora is stimulated by the host’s physical, chemical, biological, or other factors, causing abnormal proliferation of pathogenic bacteria or opportunistic pathogens that lead to bacterial diseases in *L. vannamei* [61,62]. During storage, the abundance of spoilage organisms also increases, leading to different degrees of spoilage and food quality degradation [45,46,47]. The relative abundance of potential pathogenic bacteria and spoilage organisms may help estimate their potential proliferation in farming and storage [53]. The relative abundance of potential pathogenic bacteria in the *L. vannamei* intestine varied according to different developmental stages and culture modes; however, these bacteria belonged to a relatively high abundance of bacteria [74]. In particular, *Mycoplasmataceae*, *Aeromonadaceae,* and *Shewanella* were the indicator groups in the mariculture model [75]. The relative abundance of *Vibrionaceae* and *Enterobacteriaceae* in the intestines of wild *L. vannamei* accounted for approximately 50% [52]. This result is consistent with results specifying that the proportion of potential pathogenic bacteria in some areas was high. However, the increase in potential pathogenic bacteria in the digestive system of *L. vannamei* cultured in seawater was related to the risk of disease outbreaks [52,74]. Therefore, intestinal samples of *L. vannamei* from 12 Chinese coastal cities with a high relative abundance of potential pathogenic bacteria and spoilage organisms warrants more attention. As shown in Figure 4, the relative abundance of potential pathogenic bacteria was greater than 28.2% in the GD-ZJ, ZJ-NB, JS-LYG, JS-NT, GD-DG, and TG-XQ samples and greater than 11.1% in the ZJ-NB, FJ-ZZ, JS-NT, and SD-QD samples. The breeding and preservation of *L. vannamei* shrimp from these areas should be of particular concern; although, there are many possible reasons for the observed result, including drug use, scale of farming, and water quality conditions [61,62].

Although the composition and structure of the intestinal microbiota may vary due to environmental and nonenvironmental factors, the structure and function of the normal intestinal flora play a more prominent role in the development of *L. vannamei* [60]. This relationship mainly reflects nutritional functions that provide necessary nutrition for the growth of *L. vannamei* and assists in digestion to improve the ability of nutritional metabolism, including intestinal microbes, immune function, and barrier function, which protect *L. vannamei* from the harmful effects of pathogenic bacteria and exogenous substances [76]. For immune function and barrier function, intestinal microorganisms enhance the body’s immune function and form a powerful barrier for preventing and controlling pathogenic infection with the intestinal mucosa jointly resisting the invasion by pathogenic microorganisms and maintaining the stability of the internal environment [77,78]. The main potential functions of the microbial community in the intestine of *L. vannamei* include metabolism, genetic information processing, and environmental information processing (at level 1). As previously reported, ABC transporters are the key to ensuring enzyme activity in cells. The bacterial secretion system is perceived as necessary for the secretion of protective molecules that enable bacteria to withstand harsh conditions and use DNA replication as a recovery function. These functions are crucial for the ecological balance of the intestinal microbiota and the body’s survival in harsh environmental conditions [79]. Amino acid metabolism, carbohydrate metabolism, energy metabolism, and lipid metabolism are putative functions associated with normal functioning and ensure the metabolism of organic matter by intestinal microorganisms and gain of energy by *L. vannamei* [80]. Some intestinal microorganisms of *L. vannamei* also secrete enzymes or other active substances to ensure the healthy development of the body and access to nutrients [76]. At level 3, these potential functions, including ABC transporters, bacterial secretion systems, DNA replication, cysteine and methionine metabolism, the citrate cycle (TCA cycle), and oxidative phosphorylation, showed different degrees of correlation with intestinal bacteria (Figure 6). Energy metabolism and lipid metabolism were significantly and positively correlated with most intestinal microorganisms, whereas ABC transporters and the bacterial secretion system were significantly and positively correlated with *Pseudoalteromonadaceae* and *Vibrionaceae* but significantly and negatively correlated with *Burkholderiaceae*. This result also suggests that the intestinal microbiota is actively involved in the intestinal metabolism and environmental adaptation process of *L. vannamei*. These results indicated that the intestinal microbiota of healthy *L. vannamei* respond differently to different environments and have relatively stable survival and adaptation strategies during the culture process. Therefore, energy gain and healthy growth were guaranteed despite the different culture environments. This result further demonstrated sufficient functional redundancy in intestinal metabolism in *L. vannamei*, despite some differences in the intestinal bacterial composition and structure of *L. vannamei* shrimp from 12 coastal cities in China.

In general, our investigation into the intestinal bacteria of healthy *L. vannamei* shrimp from 12 coastal areas of China detected some differences in intestinal bacterial composition and structure, especially in the composition of potential pathogenic bacteria and spoilage organisms. Although the regions were different, the potential functions of the intestinal bacterial community were similar and correlated with intestinal bacteria. However, only the intestinal bacteria of healthy *L. vannamei* shrimp were investigated. The relationship between diseases, environmental and nonenvironmental factors, changes in intestinal bacterial composition and the potential functions of *L. vannamei* found in China’s coastal areas are not known. Subsequent research will focus on this area and provide insights into the healthy cultivation and management of *L. vannamei*.

## 5. Conclusions

In this study, we investigated the intestinal bacterial community structure, composition, and differences in commercial *L. vannamei* shrimp cultivated in 12 Chinese coastal cities. For all samples, the intestinal bacterial community structure and composition varied among different regions, and the potential intestinal bacterial functions were similar and stable. These results indicate that different environmental factors in the 12 regions may play a major role in the structure of intestinal microflora of *L. vannamei*. The variation and differences between aquatic microflora in these areas over time may also be important influential factors. Therefore, it is necessary to investigate the influence of environmental and nonenvironmental factors and establish their relationship with the intestinal bacterial community in the future.

## Data Availability

All original data presented in the study are publicly available. This sequencing data can be found at: https://www.ncbi.nlm.nih.gov/sra/PRJNA731310 (accessed on 21 August 2021).

## Supplementary Materials

The following are available online at https://www.mdpi.com/article/10.3390/microorganisms9091793/s1, Table S1: Abbreviation of twelve cities spanning nine provinces along the coastline of China, Table S2: Culture conditions of *L. vannamei* along the coastline of China, Table S3: Common species of intestinal bacterial community (at the family level) of *L. vannamei* among different samples, Table S4: The top five species of relative abundance in the gut of shrimp among different regions; Figure S1: Rarefaction curves for all the samples (label: 0.03). (a) Richness, (b) Shannon–Wiener; Figure S2: Common taxa of intestinal bacterial community (at the family level) of *L. vannamei* among different samples, Figure S3: The relative abundance (at the phylum level) of intestinal bacterial community of *L. vannamei* (mean, *n* = 3); Figure S4: Predictive functional profiling (at KEGG pathway levels 1 and 2) of the bacterial communities in the gut of *L.vannamei* analyzed by Tax4Fun based on the KEGG database (mean, *n* = 3).
