# Supplementary material for: The Intestinal Bacterial Community and Functional Potential of Litopenaeus vannamei in the Coastal Areas of China
Source: Microorganisms. 2021 Aug 24;9(9):1793. doi: 10.3390/microorganisms9091793 (PMC8470311; doi:10.3390/microorganisms9091793)
Supplement: Supplementary file 1 [file microorganisms-09-01793-s001.zip › microorganisms-1348725-supplementary.pdf]

Supporting Material for

The intestinal bacterial community and functional potential of *Litopenaeus vannamei* in the coastal areas of China

Yimeng Cheng [1], Chaorong Ge [1,*], Wei Li [1] and Huaiying Yao [1,2,3]

[1] Research Center for Environmental Ecology and Engineering, School of Environmental Ecology and Biological Engineering, Wuhan Institute of Technology, Wuhan 430073, People's Republic of China

[2] Zhejiang Key Laboratory of Urban Environmental Processes and Pollution Control, Ningbo Urban Environment Observation and Research Station, Chinese Academy of Sciences, Ningbo 315800, People's Republic of China

[3] Key Laboratory of Urban Environment and Health, Institute of Urban Environment, Chinese Academy of Sciences, Xiamen, 361021, People's Republic of China

* Corresponding authors:

E-mail addresses: chaorongge@wit.edu.cn (C.G.)

Contents

Tables

Table S1: Abbreviation of twelve cities spanning nine provinces along the coastline of China

Table S2: Culture conditions of *L. vannamei* along the coastline of China

Table S3: Common species of intestinal bacterial community (at the family level) of *L. vannamei* among different samples

Table S4: The top five species in relative abundance in the gut of shrimp among different regions

Figures

Figure S1: Rarefaction curves of all of the samples (label: 0.03). (a) Richness, (b) Shannon-Wiener.

Figure S2: Common taxa of intestinal bacterial community (at the family level) of *L. vannamei* among different samples.

Figure S3: The relative abundance (at the phylum level) of intestinal bacterial community of *L. vannamei*. (mean, n = 3)

Figure S4: Predictive functional profiling (at KEGG pathway levels 1 and 2) of the bacterial communities in the gut of L.*vannamei* analyzed by Tax4Fun based on the KEGG database. (mean, n = 3).

Total:

Number of tables: 4
Number of figures: 4
Number of pages: 9

Tables

Table. S1.

Table S1. Abbreviation of twelve cities spanning nine provinces along the coastline of China

Province (Pinyin)	City name (Pinyin)	Abbreviation
Liaoning (Liáoníng)	Yingkou(Yíngkǒu)	LY-YK
Tianjin (Tiānjīn)	Xiqing(Xīqīng)	TJ-XQ
Shandong(Shāndōng)	Qingdao(Qīngdǎo)	SD-QD
Zhejiang (Zhèjiāng)	Ningbo(Níngbō)	ZJ-NB
Jiangsu(Jiāngsū)	Lianyungang(Liányúngǎng)	JS-LYG
Jiangsu(Jiāngsū)	Nantong(Nántōng)	JS-NT
Fujian (Fújiàn)	Xiamen(Xiàmén)	FJ-XM
Fujian (Fújiàn)	Zhangzhou(Zhāngzhōu)	FJ-ZZ
Guangdong (Guǎngdōng)	Dongguan(Dōngguǎn)	GD-DG
Guangxi (Guǎngxī)	Fangchenggang(Fángchénggǎng)	GX-FCG
Guangdong (Guǎngdōng)	Zhanjiang(Zhànjiāng)	GD-ZJ
Hainan(Hǎinán)	Wenchang(Wénchāng)	HN-WC

Table. S2.

Table S2. Culture conditions of *L. vannamei* along the coastline of China

Location	Longitudinal and latitudinal position (°)	pH	Sea water average temperature (℃)	Sea water quality
LY-YK	E122.306508, N40.490537	8.06	22.6	second category
TJ-XQ	E117.631196, N38.850888	8.28	23.5	second category
SD-QD	E120.276155, N36.197601	8.09	24.8	second category
ZJ-NB	E121.556852, N29.453994	8.02	24.9	third category
JS-LYG	E119.145182, N34.920879	8.07	24.4	second category
JS-NT	E121.366472, N32.369268	8.07	24.6	second category
FJ-XM	E118.212633, N24.613873	8.10	27.2	second category
FJ-ZZ	E117.756742, N23.911494	8.17	26.0	second category
GD-DG	E113.618306, N22.972389	7.72	29.5	second category
GX-FCG	E108.126094, N21.591087	8.19	27.0	second category
GD-ZJ	E110.530587, N20.970602	7.98	28.0	second category
HN-WC	E110.907513, N19.61786	8.15	30.4	second category

Table. S3.

Table S3. Common taxa of intestinal bacterial community (at the family level) of *L. vannamei* among different samples

Kingdom	*Phylum*	*Class*	*Order*	*Family*
Bacteria	*Actinobacteria*	*Actinobacteria*	*Nocardiaceae*	*Nocardiaceae*
			Nocardioidaceae	*Nocardioidaceae*
	Bacteroidetes	*Bacteroidia*	*Flavobacteriaceae*	*Flavobacteriaceae*
			Weeksellaceae	*Weeksellaceae*
	Firmicutes	*Erysipelotrichia*	*Erysipelotrichaceae*	*Erysipelotrichaceae*
	Fusobacteria	*Fusobacteriia*	*Fusobacteriaceae*	*Fusobacteriaceae*
	Proteobacteria	*Alphaproteobacteria*	*Reyranellaceae*	*Reyranellaceae*
			Rhizobiaceae	*Rhizobiaceae*
			Stappiaceae	*Stappiaceae*
			Xanthobacteraceae	*Xanthobacteraceae*
			Rhodobacteraceae	*Rhodobacteraceae*
			Sphingomonadaceae	*Sphingomonadaceae*
		Deltaproteobacteria	*Desulfobulbaceae*	*Desulfobulbaceae*
		Gammaproteobacteria	*Burkholderiaceae*	*Burkholderiaceae*
			Unknown Family	*Unknown Family*
			Pseudomonadaceae	*Pseudomonadaceae*
			Vibrionaceae	*Vibrionaceae*
	Tenericutes	*Mollicutes*	*Mycoplasmataceae*	*Mycoplasmataceae*
	Verrucomicrobia	*Verrucomicrobiae*	*Rubritaleaceae*	*Rubritaleaceae*
Unassigned	*Other*	*Other*	*Other*	*Other*

Table. S4.

Table S4. The top five species in relative abundance in the gut of shrimp among different regions

**Region**	**Feature (%)**				
LN_YK	*Rhodobacteraceae* (12.0)	*Phormidiaceae* (9.5)	*Leptotrichiaceae* (8.3)	*Flavobacteriaceae* (7.5)	*Ilumatobacteraceae* (3.6)
TJ_XQ	*Clostridiales JTB215* (25.7)	*Clostridiales Family XII* (21.5)	*Mycoplasmataceae* (21.3)	*Vibrionaceae* (6.9)	*Erysipelotrichaceae* (2.6)
SD_QD	*Bogoriellaceae* (17.4)	*Erysipelotrichaceae* (11.2)	*Flavobacteriaceae* (10.8)	*Burkholderiaceae* (6.0)	*Rhodobacteraceae* (4.6)
JS_LYG	*Mycoplasmataceae* (35.4)	*Erysipelotrichaceae* (24.5)	*Clostridiales Family XII* (13.0)	*Vibrionaceae* (9.2)	*Demequinaceae* (2.0)
JS_NT	*Mycoplasmataceae* (38.1)	*Aeromonadaceae* (4.7)	*Shewanellaceae* (4.4)	*Burkholderiaceae* (4.2)	*Rhodobacteraceae* (4.0)
ZJ_NB	*Vibrionaceae* (69.3)	*Flavobacteriaceae* (21.9)	*Erysipelotrichaceae* (2.6)	*Colwelliaceae* (1.1)	*Pseudoalteromonadaceae* (1.1)
FJ_ZZ	*Flavobacteriacea*e (14.9)	*Erysipelotrichaceae* (13.6)	*Cyanobiaceae* (13.0)	*Mycoplasmataceae* (12.6)	*Vibrionaceae* (7.6)
FJ_XM	*Desulfobulbaceae* (8.3)	*Rhodobacteraceae* (6.3)	*Marinilabiliaceae* (5.2)	*Phormidiaceae* (4.7)	*Desulfobacteraceae* (4.7)
GD_DG	*Erysipelotrichaceae* (17.8)	*Vibrionaceae* (17.1)	*Mycoplasmataceae* (15.5)	*Leptotrichiaceae* (13.6)	*Flavobacteriaceae* (5.8)
GX_FCG	*Cyanobiaceae* (14.3)	*Vibrionaceae* (9.8)	*Mycoplasmataceae* (9.8)	*Erysipelotrichaceae* (8.1)	*Chthoniobacteraceae* (4.1)
GD_ZJ	*Vibrionaceae* (85.0)	*Mycoplasmataceae* (7.2)	*Leptotrichiaceae* (3.4)	*Flavobacteriaceae* (1.7)	*Cyanobiaceae* (0.1)
HN_WC	*Mycoplasmataceae* (9.8)	*Desulfobulbaceae* (8.4)	*Flavobacteriaceae* (7.3)	*Desulfobacteraceae* (6.0)	*Leptotrichiaceae* (5.4)

Figures

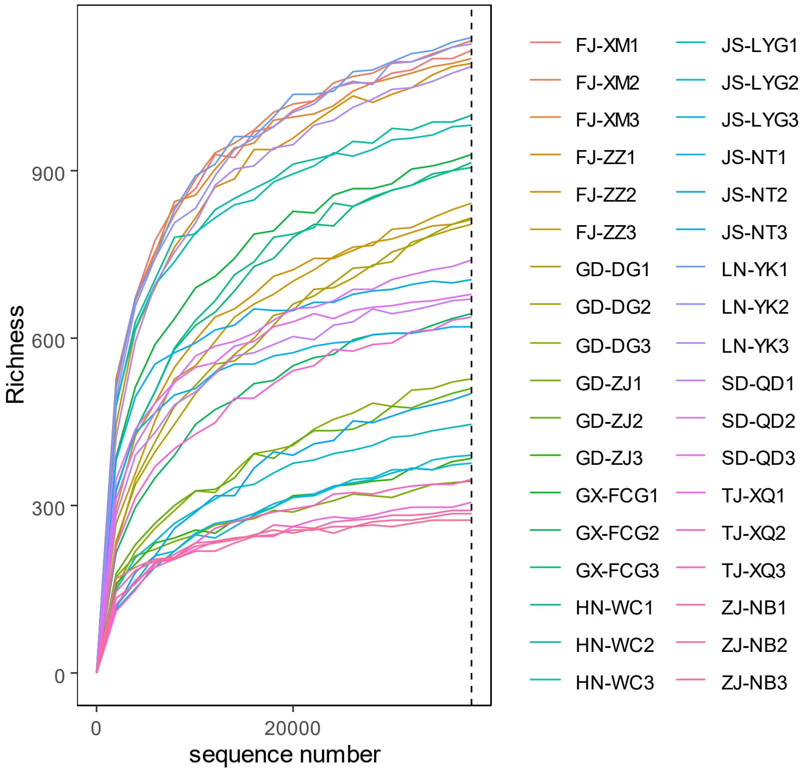

(a)

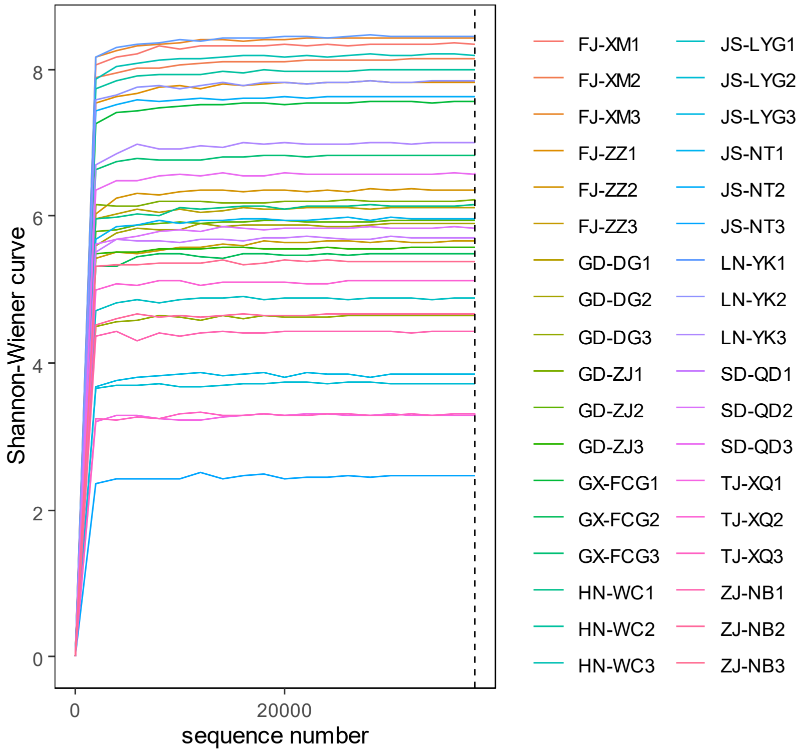

(b)

Figure S1 Rarefaction curves of all of the samples (label: 0.03). (a) Richness, (b) Shannon-Wiener.

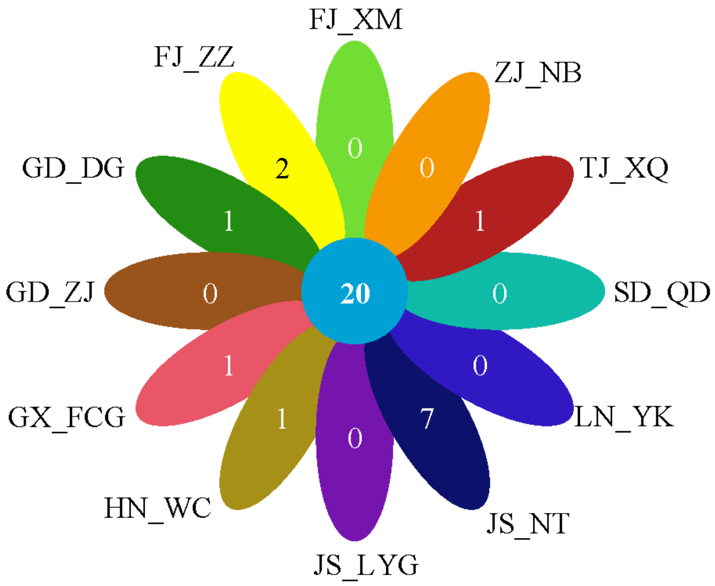

Figure S2. Common species of intestinal bacterial community (at the family level) of *L. vannamei* among different samples.

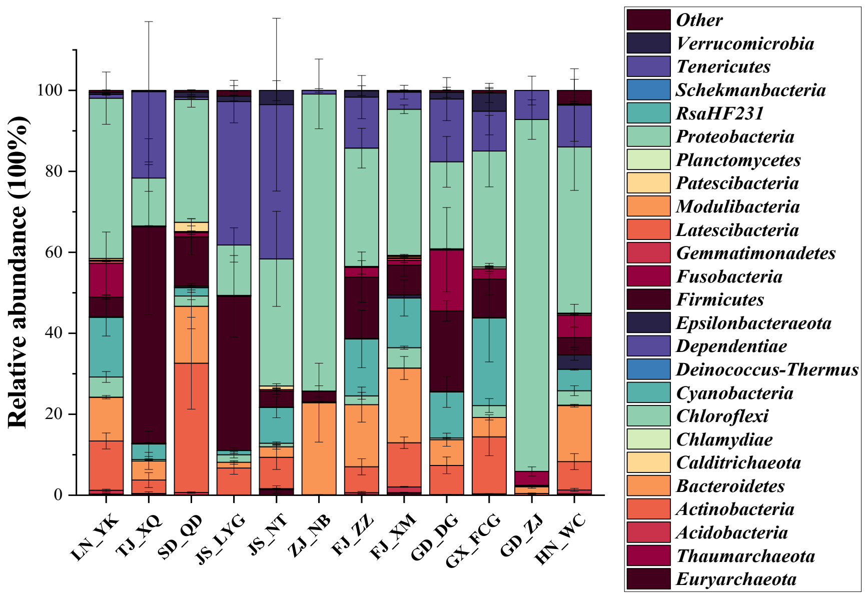

Figure S3. The relative abundance(at the phylum levels)of intestinal bacterial community of *L. vannamei*. (mean, n = 3)

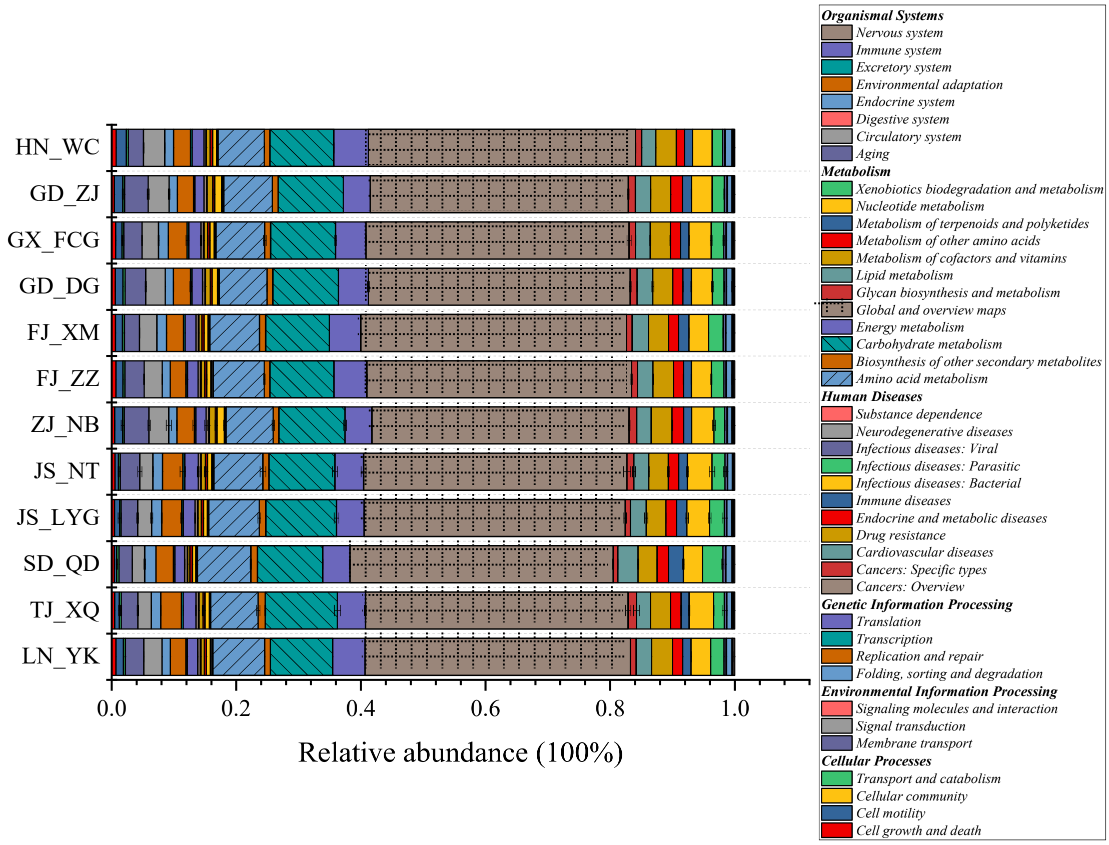

Figure S4. Predictive functional profiling (at the KEGG pathway levels 1 and 2) of the bacterial communities in the gut of *L.vannamei* analyzed by Tax4Fun2 based on KEGG database. (mean, n = 3).
